# ESL/EFL Learners' Responses to Teacher Written Feedback: Reviewing a Recent Decade of Empirical Studies

**DOI:** 10.3389/fpsyg.2021.735101

**Published:** 2021-10-29

**Authors:** Rong Yu, Luxin Yang

**Affiliations:** The National Research Centre for Foreign Language Education & Graduate School of Education, Beijing Foreign Studies University, Beijing, China

**Keywords:** ESL/EFL learners' responses, teacher written feedback, English writing, empirical studies, review, sociocultural theory of mind

## Abstract

Teacher written feedback (TWF) has long been regarded as a necessary pedagogical tool for improving the writing proficiency of ESL/EFL learners, while student responses to this feedback can often reflect its effectiveness. This paper reviews 64 articles appearing in high-ranking journals during 2010–2021 in terms of research methodology, theoretical framework and main findings. Analysis of these articles reveals few studies adopted any theoretical frameworks to examine learner responses to TWF and suggests a need for longitudinal naturalistic studies adopting mixed methods and some theoretical framework such as sociocultural theory of mind (SCT) to better explain learners' dynamic engagement in response to TWF. The main findings of these previous studies reveal the diverse responses among learners at different language proficiency levels and in various sociocultural contexts. The results of the review indicate that future research could take classroom-based mixed-method research design to investigate learner variables.

## Introduction

Teacher Written Feedback (TWF) has been recognized as an important way to improve student learning (Hyland and Hyland, 2006a,b). In recent years, although educators have increasingly put effort into exploring different types of feedback, such as peer and computer-generated feedback (Diab, 2016; Lv et al., 2021), among all types of feedback, TWF remains highly valued by English as a second/foreign language (ESL/EFL) learners regardless of their age and plays a central role in learners' language acquisition and writing development (Bitchener and Ferris, 2012; Ruegg, 2015; Lee, 2017). In second language (L2) education, how to effectively provide feedback to raise students' L2 writing proficiency has been a concern for many foreign language teachers and researchers (Sheen, 2007; Bitchener and Ferris, 2012; Kartchava and Ammar, 2014; Diab, 2015; Suzuki et al., 2019). The effective feedback may need to follow the Giving-Feedback-Guidelines which are “purpose of the feedback method,” “how the feedback method works,” and “strategies to engage with it” (Moser, 2020, p. 58). The third principle requires the engagement of the learners. Learner engagement in TWF equates to learner responses to TWF and generally involves three dimensions: affective engagement, cognitive engagement and behavioral engagement (Zheng et al., 2020b). Investigations of learner responses are not new (see Cohen and Cavalcanti, 1990; Heift, 2004; Lee, 2008). Although many studies have examined the types, effectiveness, forms and the speech functions of teacher feedback from the teachers' perspective (Lee, 2019b; Yu, 2021), students' responses to feedback have received less research attention. Reviews related to TWF have mostly focused on teacher written corrective feedback (WCF) (Chong, 2018; Li and Vuono, 2019; Mao and Lee, 2020); however, there are few, if any, reviews on ESL/EFL learners' responses to Teacher Written Corrective Feedback (TWCF) or TWF. Thus, the present review focuses on the studies that have investigated students' reactions to TWF. Furthermore, research on TWF and students' response to TWF need to consider the sociocultural context where teachers and students situate (Lee, 2014). Thus, taking a sociocultural perspective might be a way to study learners' dynamic and diversified responses after receiving TWF. As Han and Hyland (2019b) stated, “using a sociocognitive perspective can shed new light on individual variations in learner engagement by looking holistically at both the cognitive and the social aspects of language learning and language use” (p. 249). Accordingly, this review may show the need to apply sociocultural theory of mind (SCT) (Vygotsky, 1978) into the examination of learner responses to TWF.

To learn more about ESL/EFL learners' responses to TWF on L2 writing, and to provide directions for future research in this field, the following research questions guide the current review:

[1] What patterns emerge in terms of research methodology and theoretical frameworks in articles focusing on students' responses to TWF (2010–2021)?
[2] How have ESL/EFL learners responded to TWF?
[3] What factors have been found to influence ESL/EFL learners' responses to TWF?

Thus, the present review mainly focuses on TWF, and to further narrow the catchment of studies, the review is confined to ESL/EFL students' English compositions. It first briefly explains the key term **learner responses/engagement**. It then presents the methodology for this review. Results of the review are organized according to the three research questions and some new insights are also discussed from the lens of SCT.

## Learner Responses

Ellis (2008) proposed a typology of TWF in which two directions were identified. One is the teacher's provision of CF (i.e., types of CF) and the other is the students' responses to this feedback (i.e., revision). The present review focuses on the studies that investigated the latter. Ellis' framework (Ellis, 2010) used the term “engagement” rather than “students' responses” for investigations on oral and written corrective feedback. Students' responses are more than responses to revision and can be divided into three dimensions: behavioral engagement (e.g., learners' uptake or text revision), affective engagement (e.g., learners' attitudes), and cognitive engagement (e.g., learners' perceptions/views of the CF). Based on Fredricks et al. (2004) and Ellis (2010), Han and Hyland (2015) redefined the framework and established a multi-dimensional framework of learner engagement with WCF by adding sub-constructs to specify each dimension. Behavioral engagement includes both revision operations and observable strategies on raising writing accuracy and language competence. Cognitive engagement comprises the feedback processing depth, meta-cognitive operations and cognitive operations. Affective engagement also takes learners' immediate emotional responses and changes in their emotions into consideration. Later, a few studies also redefined the three dimensions in the learner engagement framework, particularly for TWCF, making only minor changes (Zhang and Hyland, 2018; Han and Hyland, 2019a; Tian and Zhou, 2020; Zheng et al., 2020a). Learner responses to some extent may be equal to learner engagement in TWF according to these studies. Although these definitions and dimensions provide directions for researchers to investigate teacher corrective feedback (both oral and written), they are not solely designed for teacher written feedback. Hence in the current review, learners' responses or engagement is defined as learners' direct responses to TWF and the following terms may be used to describe such responses, namely learner engagement, perceptions, preferences, views, attitudes, uptake, interpretation. These concepts can fall into the three dimensions that are utilized in the current review to explore learners' responses. In this review, generally, learners' cognitive responses refer to learners' noticing and processing of TWF, and the cognitive (e.g., understanding) and meta-cognitive strategies (e.g., evaluating and planning) that learners may have used. Behavioral responses were the directly observable and traceable behaviors after learners receiving TWF (e.g., revision). Attitudinal responses encompass the affect (e.g., feelings and emotions), judgment (e.g., personal and moral), and appreciation of TWF.

The studies on indirect responses (i.e., the effect of TWF, such as improvement in writing accuracy) are not included in the current review. The effect of TWF appears to be one aspect reflecting learners' behavioral responses; however, in these reviewed studies, learners' responses, which may be regarded as a passive response, is not the primary research focus; instead, studies focusing on the effects simplify the relationship between teachers and learners in which teachers are the only provider of feedback and learners are the only receiver. The results focusing on this aspect could only inform researchers and educators whether this type of feedback might work for certain group of students and naturally ignore the learners' thoughts behind their motive; thus, the focus of this review is on learners' responses.

## Methodology

To address the three research questions, this paper reviews the empirical studies on ESL/EFL learners' responses to TWF published during 2010–2021.

Guided by three research questions, a series of inclusion criteria for articles was established to identify journal articles related to TWF over the past 10 + years (Table 1). Specifically, only articles written in English containing empirical research published in SSCI-Index journals from January 2010 to September 2021 (the last date for the data searching was September 9th) were reviewed. In the articles, TWF (handwritten and/or electronic) on L2 writing were provided by the participating teachers and/or researchers. ESL/EFL learners' responses toward TWF, the theoretical framework and the methodology of the research were also clearly stated and analyzed. Only articles in high-ranking journals were included.

**Table 1:** Inclusion criteria in the searching phase.

**Criteria**	
**Year**	**2010.1–2021.9**
Feedback providers	Teachers and/or researchers
Feedback receivers	ESL/EFL learners
Feedback type	Teacher written feedback (handwritten and/or electronic)
Language	Written in English
Publications	SSCI journals
Research	Empirical research
Articles	Full research articles (excluding conference articles, book chapters)

To identify the journal articles that met the inclusion criteria, 10 academic databases were selected including Oxford Academic Journals, Cambridge Core, ScienceDirect, Web of Science, Taylor and Francis Online, EBSCOhost, SAGE, ERIC, SpringerLink, and Wiley Online Library. Multiple databases ensure that more relevant articles can be included. The keywords used for the literature search were: teacher written feedback + English as a second language (ESL)/English as a foreign language (EFL)/L2 writing. During the search, the SSCI journals were checked through the Master Journal List in the Web of Science platform. The initial data searching included journal articles published between January 2010 and April 2021. Informed by the titles and abstracts of the articles, a total of 240 articles were first identified. To update the data, a second round of data searching was conducted to search the articles published between January 2021 and September 2021. The second search from January 2021 was mainly due to the lack of month selection in advanced research of some databases; thus, ensuring more relevant papers were included. After the second search, 56 articles were found. Altogether 177 articles were found (e.g., Xu, 2021; Zhang and Cheng, 2021). Next, articles that were mistakenly included in both search results were eliminated which left 160 articles. Using Mendeley, software designed for organizing research papers, all articles' publication dates were checked again since not all databases provided the choice to choose the month to search for articles. Besides 17 excluded articles that were discovered to be mistakenly selected in searching phrase, 96 articles were excluded mainly due to their research focus or content inconsistent with this review's purpose and paper selection criteria. These excluded journal articles were coded into eight primary themes: effects of feedback (*N* = 47); wrong year (*N* = 1); not ESL/EFL learners as participants (e.g., Spanish) (*N* = 5); a survey unrelated to any specific L2 writing courses or experience of receiving TWF (*N* = 5); unclear methodology description (*N* = 3); focusing on teacher written feedback practices or oral feedback or peer feedback (*N* = 9); automated writing evaluation (*N* = 2); others (little relevant to L2 writing or about ESL/EFL learner responses to TWF) (*N* = 24). Notably, a certain number of articles related to effects of teacher written feedback were left out while some remained to be reviewed, because the primary or secondary focus of those reviewed articles were learners' responses to TWF.

After a more careful examination of the remaining 160 articles regarding the publication dates, introduction, research aims, questions, methodology and findings to eliminate irrelevant articles that might be mistakenly chosen in the searching phase, the research obtained the final sample of 64 relevant empirical studies highly related to ESL/EFL learners' responses to TWF on L2 writing as the body of research for the synthesis.

Using a content analysis approach, authors reiteratively read the selected articles and coded them according to their themes and foci. With the help of Excel, the articles were then coded in response to the three research questions. The coding scheme for the research methodology was comprised of 10 categories of variables: research methodology, methodological designs, being naturalistic or not, case study or not, research country, educational level, course, duration, participants, and methods. As the authors tried to define the nature of the studies, it was found that not all the studies were purely qualitative, quantitative or mixed; therefore, the research methodology and methodology designs were coded based on Riazi et al. (2018) and Hyland (2016), respectively. Research methodology codes thus included qualitative, quantitative, mixed, eclectic (QUAL + quan, QUAN + qual, QUAL + QUAN); methodology designs codes were auto-ethnography, experimentation, case studies, quasi-experiment, and other designs. Some coded selected journal articles are presented in Appendix I.

## Results

The results of the review are presented in the following three sections concerning the research methodology, theoretical framework, and the synthesis of key findings from the selected articles.

### Research Methodology

The results show that among the 64 articles, 34 were qualitative, seven were quantitative, six were mixed design, 10 were eclectic (QUAL + QUAN), six were eclectic (QUAN + qual), one was eclectic (QUAL + quan) (see Table 2). Among the qualitative studies, more than half of the articles (*N* = 19) described case studies and only one was auto-ethnography. More than half of the studies (*N* = 36) were categorized as other designs in which qualitative studies' number still outweighed the other types.

**Table 2:** Coding frequencies.

	**Auto-ethnography**	**Experimentation**	**Case studies**	**Quasi-experiment**	**Other designs**	**Total**
Qualitative	1		19		14	34
Quantitative		4		1	2	7
Eclectic (QUAL + quan)					1	1
Eclectic (QUAN + qual)		2			4	6
Eclectic (QUAL + QUAN)		1			9	10
Mixed					6	6
Total	1	7	19	1	36	64

The number of naturalistic studies (*N* = 52) (i.e., classroom-based studies) was approximately four times the number of experimental studies (involving interventions). This may reflect the recent trend of researchers investigating teachers' feedback practices in real classroom contexts. Studies have shown that interventions are very useful when the effect of TWF is investigated, especially efforts to make comparisons between different feedback types (Bitchener and Knoch, 2009a,b, 2010). In the present review, intervention studies using experimental or quasi-experimental designs were also with the examination of the feedback effects, such as the comparisons made between three types of directness forms (i.e., direct speech acts, indirect speech acts, and hedging) (Baker and Bricker, 2010), two feedback modes (i.e., computer-mediated and computer-generated feedback) (Sherafati et al., 2020), three focuses of comprehensive WCF (i.e., accuracy, syntactic complexity, and fluency) (Zhang and Cheng, 2021), and two feedback types (i.e., indirect coded correction feedback with and without short affective teacher comments) (Tang and Liu, 2018). Although these studies and other intervention studies may not take the learners' responses as their main research purposes, the findings related to responses were also important in these reviewed studies.

As Table 3 shows, more than 85% of the studies (*N* = 55) were conducted at the tertiary level including both English major and non-English major ESL/EFL learners; very few were in secondary and primary schools. In tertiary education, the participants in most of the reviewed studies were undergraduates (*N* = 45); few studies (*N* = 10) had postgraduates as their participants (Rivens Mompean, 2010; Storch and Wigglesworth, 2010; Mirzaee and Hasrati, 2014; Kim and Kim, 2017; Xu, 2017; Green, 2019; Zheng et al., 2020b; Anderson, 2021; Pitura, 2021; Saeed et al., 2021). The number of studies situated in China was 24. In the studies located outside China where English is the first language in local areas, such as the U.S., Australia and the UK, the participants were mainly international students who were ESL/EFL learners. A few studies were in Japan, Spain, Canada (Quebec), Iran, South Korea, Turkey and another 13 countries all of which share a similar English language learning context.

**Table 3:** Research location and learners' educational levels.

**Country**	**Educational level of the student participants**			**Total**
	**Primary**	**Secondary**	**Tertiary**	
China	1	2	21	24 (37.5%)
U.S.			9	9 (14.1%)
Japan			3	3 (4.7%)
Spain	2	1		3 (4.7%)
UK			3	3 (4.7%)
Canada		1	1	2 (3.1%)
Iran			2	2 (3.1%)
South Korea		1	1	2 (3.1%)
Turkey			2	2 (3.1%)
Other countries			13	13 (20.3%)
Unknown	1			1 (1.6%)
Total	4 (6.3%)	5 (7.8%)	55 (85.9%)	64 (100%)

*The other 13 countries were Australia, Costa Rica, France, Germany, Malaysia, Nepal, Norway, Pakistan, Poland, Saudi Arabia, Sweden, Thailand, and Yemen, which had one paper each. Only one article did not state the research country*.

The data collection methods in the 64 articles (Table 4) were mainly determined by the specific aims of the research, although all the studies shared the same focus—ESL/EFL learners' responses to TWF on English writing. The research methods covered in the studies included: semi-structured interviews with the teacher(s), students(s); learners' verbal reports (e.g., think-aloud protocols); questionnaire; class observation (e.g., field notes); and the content analysis of: written reports (e.g., reflective accounts, written verbalization), writing drafts, peer dialogues, teacher written feedback, peer feedback and/or automated feedback, questionnaire, teacher-student writing conferences, class documents (e.g., lesson plans, textbooks, grading rubrics, writing prompts, the syllabus, handouts, teaching slides), and other documents (response times, feedback requests and screen recordings) (see Baker and Bricker, 2010; Maas, 2016; Bakla, 2020).

**Table 4:** Data collection methods.

**Methods**	**Number**
Semi-structured interviews with teacher(s)	9
Semi-structured interviews with student(s)	39
Verbal reports (e.g., think-aloud protocols)	10
Written reports (e.g., reflective accounts, written verbalization)	13
Peer feedback	5
Automated feedback	6
Questionnaire	25
Teacher-student writing conferences	7
Class observation (including field notes)	10
Class documents	8
Peer/pair dialogue recording	9
Other documents	3

Although the data analysis was not the focus of the present review, most of the studies used qualitative or qualitative-dominant methods and coded the various datasets; thus, only the methods for data collection are presented here.

### Theoretical Frameworks

To investigate the learners' responses, most studies (*N* = 39) did not explicitly use any theoretical framework to inform their studies; only 26 studies were based on a certain framework.

To investigate learner engagement, nine qualitative studies (including eight cases studies) adopted and adapted the framework of learner engagement proposed by Fredricks et al. (2004) and Ellis (2010) and critically integrated the findings of some later studies as the analytical or conceptual framework to analyze learners' responses to TWF. Surprisingly, all nine studies were located in China's tertiary educational context, five of which were case studies focusing on how Chinese undergraduates responded to TWCF (Han and Hyland, 2015; Zheng and Yu, 2018; Han, 2019; Zheng et al., 2020a; Han and Xu, 2021). For example, Han (2019) took an ecological perspective to examine learners' engagement with TWCF and the close interactions between L2 learners and their surrounding environment. Using the learner engagement analytical framework, the other five studies explored learners' responses to supervisor feedback on their masters' thesis (Zheng et al., 2020b), collaborative writing and revision (Zhang Z., 2021), two feedback sources (i.e., both TWF and automated feedback) (Zhang and Hyland, 2018) and three feedback sources (i.e., TWF, automated feedback and peer feedback) (Tian and Zhou, 2020). As stated above, the framework of learner engagement was adapted to different but limited research foci.

Six reviewed studies based their research on sociocultural theory of mind, a theory arguing that learner development is a mediating process from object-regulation and other-regulation to self-regulation (Vygotsky, 1978). These studies were also located in various universities, with three of them being case studies. One case study, also an auto-ethnography study, described a Chinese-L1 female PhD candidate's experience with a white New Zealand supervisor's feedback based on the Zone of Proximal Development, a concept in SCT (Xu, 2017). Another case study, Storch and Wigglesworth (2010), investigated the effects of peer discussion on learners' engagement with TWF through the lens of SCT. The third case study, Saeed et al. (2021), analyzed two female Malaysian postgraduates' behavioral responses (i.e., proposal writing revision) to supervisory feedback. Based on the sociocultural theory of learning, Kim and Emeliyanova (2021) tried to understood learners' engagement with teachers' indirect WCF in two different learning environments (i.e., collaborative revision and individual revision) and the influence of the environments on learners' writing accuracy. Two other studies integrated three theoretical frameworks to support their research. Mohammed and Alharbi (2021) used the mediation concept together with social constructivist theory and feedback dialogue models and frameworks to analyze learners' responses in the feedback dialogue and influencing factors. By taking a grounded theory approach that could be used when the responses were not analyzed within any theory that aimed at only one aspect or subcategory of the engagement framework, Mahfoodh (2017) also investigated EFL learners' emotional responses to TWF combining three theories (i.e., the cognitive process theory of writing, the socio-constructivism theory of learning, the sociocultural theory of learning) to inspect the data and establish a model to depict the complex relationship between TWF, students' emotional responses, and students' success of revisions.

The remaining 11 studies also chose university students as their main student participants to investigate learners' responses to TWF by linking them to various theories, covering at various responses in their studies. The theories included: the noticing hypothesis and output hypothesis (Hanaoka and Izumi, 2012); a taxonomy of academic emotions (Han and Hyland, 2019a); self-regulated learning (Xu, 2021); a tripartite definition of written feedback (Chong, 2019); dialogism (Turner, 2021); systemic functional linguistics (Zhang X., 2021); ecology (Lee et al., 2021); L2 socialization theory (Anderson, 2021; Pitura, 2021); a socio-constructivist approach (Rivens Mompean, 2010); the framework of learning-oriented language assessment (Kim and Kim, 2017); and a second language socialization theoretical framework (Anderson, 2021).

### Students' Responses to Teacher Written Feedback

Learners' responses vary when they are at different language proficiency levels and writing ability. Thus, how learner respond to TWF was reviewed according to the educational level they were at when they were participating in the studies from the three-dimension learner response model. In the following sections, the responses are revealed in the three dimensions.

#### Learners' Responses in Tertiary Education

##### Attitudinal Responses

Based on the findings of the reviewed studies, learners were found to commonly hold a positive welcoming attitude toward TWF, including learner-driven feedback (Maas, 2016) and teacher-dominant feedback given out of teachers' subjective judgment, such as written corrective feedback (Han, 2017; Xu, 2021). More specifically, learners' attitudes were reflected in their preferences toward diverse TWF.

Regarding feedback mode, ESL learners preferred electronic written feedback (computer-mediated feedback/e-feedback) (Chong, 2019), even when it was compared with digital audio and screencast feedback (Bakla, 2020). Although e-feedback is a relatively new mode of feedback appearing with the advancement of technology, EFL learners in Japan preferred handwritten feedback which was regarded as a cultural relic and custom, hoping it could be provided within one manuscript (Elwood and Bode, 2014). Face-to-face feedback was also more welcomed by EFL undergraduates in an English for academic purpose (EAP) program in a U.S. university in spite of the potential benefits of e-feedback (Ene and Upton, 2018). Six MA students who majored in ESL teaching argued that they preferred both oral in-class and written online out-of-class feedback on their master' thesis (Pitura, 2021).

The sources of feedback are fundamentally three: TWF, peer feedback and automated feedback (computer-generated feedback). Learners' preference was revealed mainly through comparison between these three sources. When comparing teacher and peer feedback, Chinese EFL learners valued both sources, but they attached greater importance to TWF for its higher perceived effectiveness and help in revision (Lam, 2013; Tsao et al., 2017). When TWF was compared with automated feedback, such as Criterion, ESL learners' preferences showed a divergence that the trust and appreciation given to these two vary (Dikli and Bleyle, 2014). However, learners' preference was dynamic and could change at different stages of the writing process. For instance, Tian and Zhou (2020) found that in three essay cycles, one EFL learner initially strongly valued peer feedback and hated teacher feedback in the first two cycles, but he regarded TWF as the most helpful source in the third cycle. A similar preference change between automated feedback and TWF could also be found in this study. In Iranian EFL learners' writing experience, learners either support computer-mediated TWF or computer-generated feedback (Sherafati et al., 2020). In effect, preference was not always extreme and learners also expected to receive the combination of two sources, namely both teacher and peer feedback (Tsao et al., 2017).

Learners' preference for feedback type was related to the directness, focus, explicitness, correction and comments with specific features. Some learners preferred direct detailed feedback (e.g., direct corrective feedback) (Elwood and Bode, 2014; Niu et al., 2021) and others preferred indirect written feedback (e.g., indirect coded correction feedback or such feedback along with short affective comments) (Kim and Kim, 2017; Tang and Liu, 2018; Mujtaba et al., 2020). Similarly, no definite trend was discovered between the choice of selective (focused) feedback (Ferris et al., 2013) and comprehensive (unfocused) feedback (McMartin-Miller, 2014; Sherafati et al., 2020; Lee et al., 2021; Zhang and Cheng, 2021), but feedback that pointed out learners' L2 writing shortcomings (Pitura, 2021), was informative on the content (Bastola and Hu, 2021) or provided metalinguistic explanations for grammatical and orthographic errors (Zhang et al., 2021) was preferred by a certain number of English language learners. Also, learners seemed to prefer WCF in general as written comments (Ene and Kosobucki, 2016), or with the feature being coded (Han, 2019), or being explicit and overt compared with three other WCF types (underlining, error coded, metalinguistic explanation (Zhang et al., 2021).

The time of feedback has also been investigated recently. ESL/EFL learners usually receive asynchronous feedback and students' attitudinal responses vary. If synchronous and asynchronous TWF were compared, learners might prefer engaging in synchronous TWF, as shown in Ene and Upton (2018). The color in which feedback was given, however, seemed to be an issue of little concern, although Elwood and Bode (2014) revealed that male learners showed a slight preference for red over blue.

Within the attitudinal responses, emotional responses to TWF were significant. Various positive, negative and neutral emotions experienced by learners after receiving TWF were observed. These evoked emotions were either individual or social. Individual emotions were emotions specifically for the learners themselves. Positive individual emotions included feeling validated and respected, feeling of happiness, satisfaction, being pleased, and gaining reassurance while negative individual emotions comprised disappointment, feelings of being misunderstood, being mistreated, anxiety, hopelessness, upset, uncertainty, worry, guilt, and self-consciousness (Han and Hyland, 2015, 2019a; Mahfoodh, 2017; Ene and Upton, 2018; Han, 2019; Zheng et al., 2020a,b; Anderson, 2021). A neutral individual emotion was relief (Han and Hyland, 2015, 2019a). Social emotions are emotional responses toward teachers and TWF. For teachers, learners were found to mostly show respect, trust, awe, and gratitude (Mahfoodh, 2017; Zhang and Hyland, 2018; Han and Hyland, 2019a; Tian and Zhou, 2020; Zheng et al., 2020a,b; Zhang Z., 2021), which can be categorized as positive emotions, although distrust may also happen (Li and Curdt-Christiansen, 2020; Zheng et al., 2020a). Emotions to TWF went in three directions. Positively, learners treated the feedback with appreciation, welcome, high value and contentment (Ene and Kosobucki, 2016; Zhang and Hyland, 2018; Zheng and Yu, 2018; Han and Hyland, 2019a; Zheng et al., 2020a; Zhang Z., 2021). Negatively, learners also experience emotions such as doubt, disagreement, rejection, slightly shocked, frustration, surprise, confusion, all of which resulted in little enthusiasm to revise (Mahfoodh, 2017; Zhang and Hyland, 2018; Zheng and Yu, 2018; Han, 2019; Han and Hyland, 2019a; Li and Curdt-Christiansen, 2020; Zheng et al., 2020a; Anderson, 2021). Social neutral emotions were mainly the acceptance of feedback (Mahfoodh, 2017). Learners' emotions were not steady all the time and could change from negative to positive (Han and Hyland, 2015) or to neutral (Zheng et al., 2020a) or from neutral to negative (Han and Hyland, 2019a). In sum, it was a dynamic process.

##### Cognitive Responses

Cognitive responses were split into three subcategories: noticing, processing, and cognitive/metacognitive operations. Noticing refers to the identification of the types of TWF, and information conveyed through feedback that might be useful for revision or individual language proficiency development.

Regarding noticing content, learners' attention was mainly on the lexical level, such as erroneous form (Yang and Zhang, 2010), and solutions for revision by comparing individual drafts with models or reformulated essays provided by teachers. The noticing speed and accuracy was also tested in one of the reviewed studies (Baker and Bricker, 2010) showing there was better speech and accuracy in positive feedback (compared with negative feedback) and direct feedback (compared with hedged comments), and better accuracy after negative comments that required text correction.

After noticing, learners usually need to process the feedback and perhaps hidden messages (Hyland, 2013); thus, different cognitive operations would be utilized. Cognitive operations are the cognitive or meta-cognitive strategies used in processing TWF. Cognitive operations mentioned in the reviewed articles including conceptualizing on details, reasoning, memorizing, activating previous knowledge, clarifying, questioning, confirming, and justifying (Han and Hyland, 2015; Buckingham and Aktuǧ-Ekinci, 2017; Saeed et al., 2021). Meta-cognitive operations mainly concerned having planned steps to deal with teacher feedback, intentions and plans to make revisions, providing a correct form/response, informing of making a revision, prioritizing, evaluating, monitoring, letting it go and moving on (Han and Hyland, 2015; Zhang and Hyland, 2018; Zheng et al., 2020b; Saeed et al., 2021). With the assistance of these operations, learners can process the feedback at different depths, either high or low. For instance, reformulation, a form of written corrective feedback, might require higher cognitive engagement than direct corrections (Kim and Bowles, 2019) while it was found to be lower than the editing processing (Storch and Wigglesworth, 2010). The cognitive engagement was different in processing various feedback types. For example, the processing difficulty level ranked from high to low was hedged comments, indirect comments and direct comments (Baker and Bricker, 2010). Processing is a dynamic process but it still has a result. According to the findings of the reviewed research, the outcome of processing included three layers of comprehension, namely understanding, misunderstanding (partially understanding) and no understanding.

##### Behavioral Responses

Attitudinal responses and cognitive responses are highly related to internal responses and behavioral responses are thus external responses. Behavioral responses reflected learners' revision operations, and other relevant sociocultural learning behavioral responses. Like cognitive engagement, behavioral engagement also requires revision operations. This review revealed that revision operations encompassed revision strategies and external resource utilization. Revision strategies generally were found to be dichotomous: change (modification) or no change (null text revision). A learner could simultaneously make use of all seven revision operations (i.e., correction, no correction, deletion, substitution, addition, rewriting, reorganization) in revising drafts (Zhang and Hyland, 2018). As for external resources, ESL/EFL learners mainly sought help from peers (Han and Hyland, 2015; Bader et al., 2019) and learning materials, such as online dictionaries (e.g., Youdao) and spelling check of word processing software (Han, 2019; Tian and Zhou, 2020), and a grammar book (Zheng et al., 2020a) while seldom asking additional help beyond TWF from their teachers.

Behavioral responses also reflected the uptake of feedback during revision, or the effort put into the revision. Feedback uptake in revision means the behavior of using feedback in revision and it varied with the types of TWF. Saeed et al. (2021) discovered that expressive feedback, such as praise, was a typical example that required no effort in revision while directive feedback's uptake in revision was relatively much higher than expressive and inferential feedback. Indirect coded correction feedback was also found to elicit learners' more revision efforts in the error reduction and improvement of writing performance. However, if the error correction was overt that most errors were explicitly identified, the uptake might be high but with limited engagement and the revision would become mechanic and less meaningful (Zhang and Hyland, 2018). Moreover, the uptake of TWF, peer feedback, and automated feedback in revision also varied from learners to learners as revealed in Tian and Zhou (2020). Learners also showed opposite incorporation tendencies when it concerned meaning-related and surface-level TWF.

In one of the reviewed studies (Baker and Bricker, 2010), the revision speed and accuracy was assessed. Direct comments were found to have the highest accuracy when revised although the revision speed with this type of comments appeared to the slowest. However, revisions after receiving indirect comments were faster than direct ones but with low accuracy. With different revision operations, feedback uptake and revision speed and accuracy, the outcome of the revision was either successful or unsuccessful (Zheng and Yu, 2018; Bader et al., 2019).

Social behaviors were also elicited by TWF. One EFL learner, Sissy, experienced feelings of being “misunderstood, mistreated, and unfairly critiqued” after receiving negative TWF; thus, she chose to marginalize herself in the college community that she belonged (Mirzaee and Hasrati, 2014). Students also tried to utilize feedback for their learning reflection.

#### Learners' Responses in Secondary Education

Five journal articles were highly relevant to learner's responses in secondary education. The results show that learners' responses were dynamic and varied. Learners held either positive or negative attitudes toward TWF (Kang, 2020); however, attitudes could change from negative (e.g., blame) to positive (praise) when learners received direct and indirect WCF (Simard et al., 2015). Regarding emotions, learners from an English medium secondary school in China were satisfied with TWF, particularly its range and depth (Lee et al., 2013). However, some learners might feel discontent with WCF when the right answers were provided (e.g., all the errors were corrected by the teacher) (Simard et al., 2015) or bored when they tried to engage in a particular type of TWF, model essays (García Mayo and Labandibar, 2017). Learners' cognitive responses included noticing and processing. Learners noticed the gaps between their written drafts and TWF and noticing primarily focused on lexical issues (García Mayo and Labandibar, 2017). Other than gap noticing, learners also noticed new ideas, expressions, solutions for previous and new writing problems by comparing models and self-drafts (Coyle et al., 2018). As for processing, indirect WCF seemed to be more difficult to interpret than direct WCF (Simard et al., 2015). Learners' behavioral responses were not the focus in this educational level studies. The result demonstrates their willingness to rewrite (Simard et al., 2015) and uptake the perceived features in the model essays (García Mayo and Labandibar, 2017).

#### Learners' Responses in Primary Education

Few articles (*N* = 4) were found highly relevant to the review topic. Mak (2019), one of the reviewed studies' results showed the different preference for WCF in two writing classes. One class preferred direct comprehensive WCF and another class preferred coded WCF. However, one class changed its preference from direct comprehensive feedback to coded WCF after the class adopted innovative feedback approaches. This study also revealed learners' preference on the color (i.e., less red ink) and clarity (i.e., less mess) of the feedback. Learners' cognitive responses were found mainly about noticing and processing. Through analyzing learners' pair discussions and interviews with learners, the current reviewed studies found that lexical features were the focus of the noticing (Coyle and Roca de Larios, 2020), although other features (e.g., content, sentential features) might also be noticed to different extent (Luquin and García Mayo, 2021). Moreover, results concerning learners' behavioral responses primarily revealed the three revision operations (i.e., selective changes, unacceptable changes, no change) that learners had used related to the sentential level (Cánovas Guirao et al., 2015) and some learners were able to self-correct the errors if manageable TWF (i.e., coded and focused feedback) were provided (Mak, 2019).

### Factors Influencing ESL/EFL Learners' Responses

In this review, ESL/EFL learners' responses to TWF were closely related to various sociocultural factors. Inspired by SCT, although the influencing factors have been called individual/learner factors and contextual factors in other studies (Ferris et al., 2013; Han, 2019; Zheng et al., 2020a), this review used two new terms to describe these two categories respectively: intra-factors and inter-factors.

#### Intra-factors

Intra-factors are factors that can be controlled and regulated by the learners themselves. Personal factors may be factors limiting learner's engagement, including gender and age. Males showed a strong preference for red ink and minimal amount feedback while females cared little about the color of the feedback and preferred detailed feedback (Elwood and Bode, 2014); further, the younger the learners were, the more psycholinguistic constraints they might have (Coyle and Roca de Larios, 2020). Attitudinal-related factors were also significant for their effect on learners' preference (e.g., preference for writing), attitude (toward the writing tasks or to the feedback) and emotions (e.g., trust/distrust in the teacher, feedback, satisfaction with the first draft). Notably, language learning enjoyment also played a significant role in influencing learners' attitudinal and behavioral responses (Zhang et al., 2021). Another group of intra-factors were found to be willingness-related. This group of factors included motivation, willingness to make or avoid making mistakes, learner beliefs in their teachers' authority or responsibility, dependence on TWF and personal feedback focus (e.g., error focusing), and learners' eagerness to make changes. Capacity-related factors were also found to be significant. Most capacity-related factors were related to learners' language ability or language proficiency level, such as the ability to revise, linguistic competence (e.g., lexical or grammatical knowledge), processing capacity (particularly for younger learners), feedback literacy and the learning strategies learners use. Learners' learning experiences revealed some experience-related factors, namely learners' previous experience with L2 or feedback (e.g., failure) and revision achievement. Other intra-factors included self-consciousness-related factors (e.g., confidence) (Ferris et al., 2013) and goal-related factors (e.g., learning goals, expectation) (Han and Hyland, 2015; Turner, 2021).

#### Inter-factors

Inter-factors are related to the environment; thus, learners have weak control of them. Inter-factors can be further divided into macro-factors and micro-factors. Macro-factors include four sub-categories.

The first category concerned feedback, which could also be said to be inter or intra, which depending on the influence occurred within or out of one type of feedback. Feedback-inter-factors are highly concerned with the feedback mode (with or without computer assistance), time (how fast learners could receive TWF, quantity (the amount of feedback), types (focused or unfocused, specific/detailed or general/summative, explicitness, directness, WCF, affective comments), usefulness/effectiveness, applicability (e.g., value for revision), form (e.g., color), place (within one page or in separate manuscripts), affective effect (being encouraging or discouraging), feedback language use (e.g., language complexity), depth, clearness, seriousness (i.e., how serious the error was for learners), correctness (e.g., whether the feedback was right or wrong), supportiveness and most importantly comprehensibility. Feedback-inter-factors were mainly source-related factors, i.e., influence from other feedback sources (i.e., peer feedback, autonomous feedback) together with TWF.

Another important factor was the teacher. Results showed that a healthy and positive teacher-student relationship could promote learners' positive engagement. Teachers' classroom instruction was also very important. For instance, shared understanding on feedback giving rationales (Mak, 2019) and the thoughts of the importance of the self-correction injected by teachers in class (Simard et al., 2015) seemed to be able to enhance learners' engagement.

The third category was activity-related factors. The task's nature might influence learners' focus, such as the close relation between the meaning-focused tasks and young learners' lexis focus when processing TWF. In some feedback processing tasks, young learners were required to take notes (i.e., write down anything they noticed when processing the TWF), which seemed to be another effective way to engage learners (García Mayo and Labandibar, 2017). The accessibility to TWF offered to peers gave learners' opportunities to engage more. Also, the use of correction code sheets also resulted in more successful revision responses (Buckingham and Aktuǧ-Ekinci, 2017).

However, some activities disengaged learners as they responded to TWF. No requirement for revision, busy learning schedule, and the activities that were too long and boring might lead to limited engagement. Peers are of no less importance than teachers. When learners encountered difficulties in processing TWF, some of them would seek help from peers and the in-class peer discussion would also increase learners' engagement. However, activities that seemed to be encouraging might become discouraging by causing frustration, such as rebuttal writing tasks (Man et al., 2020).

Macro-factors are culture-related factors and environmental factors. Apart from the abovementioned cultural influences in Japan education, Chinese learners also confronted conflicts between western and eastern culture (e.g., Confucianism) which impacted their responses (Xu, 2017).

## Discussion and Implications

The present review aimed to investigate ESL/EFL learners' responses to TWF in the last decade (i.e., 2010–2021) as well as shed light on possible future trends. The results are discussed with the help of the sociocultural theory of mind perspective; implications are also provided for future research regarding three aspects: methodology, theoretical frameworks, and students' responses to TWF.

### Methodology

Most reviewed articles described qualitative or qualitative dominant studies; case studies were especially prominent, which indicates recent trends. Qualitative studies play a dominant role in investigating students' inner voices from various perspectives, which may be the reason there were few quantitative and mixed methods studies. From a sociocultural perspective, it is important to understand what makes TWF (e.g., CF) effective in learners' L2 development (Bitchener and Storch, 2016). Thus, it seems that studies with learners' self-reports can deeply reveal the complex connections between TWF and student engagement. A large number of studies were naturalistic, which indicates a focus on the sociocultural context of learners' responses. As SCT contends, the environment is the source of learners' mental development; thus, perhaps the more naturalistic the research context is, the more realistic the responses would be and the more reliable data are for shedding light on learner responses. TWF is not a static response to students' drafts. It is “always situated in an ongoing dialogue between teachers and students” (Hyland and Hyland, 2006c, p. 213). In other words, TWF is co-constructed in student-teacher interactions and its mediating role needs to be studied in various naturalistic contexts.

Participants in the reviewed studies were mostly undergraduates with few studies investigating students in secondary or elementary schools or postgraduates. Since students receiving elementary and secondary education are often beginners or low-intermediate ESL/EFL learners, how TWF scaffolds and mediates their writing and promote their language development is worthy of investigation. Even though postgraduates have reached a higher level of language proficiency, their language ability still differs from their peers who are native English speakers. The mediating role of the TWF also needs more attention. Leaners can also be classified according to their learning styles, beliefs, motivation, gender, preference of different types of feedback (e.g., direct, indirect, metalinguistic, focused or unfocused) by filling out a learner profile sheet (López et al., 2018) at the beginning of the research. Students' responses are dynamic and may change frequently by interacting with various sociocultural factors (e.g., classmates, teachers). Environment is the source of development, and ESL/EFL learners develop their L2 or foreign language mainly through environment-person interaction (Lantolf and Poehner, 2014). From an activity theory perspective, sociocultural factors cooperate with each other to ensure the successful operation of the learning activity (Yu, 2013). As a “highly complex concept,” “learner engagement” is both “a process that can change during the course of time” and “more a process which can result in an outcome” (Moser, 2020, p. 13–14). In other words, learners' responses are dynamic and need to be studied based on this nature. More longitudinal studies are needed to investigate the change of learners' responses as they produce multiple drafts in more than one cycle of feedback and revision (Tian and Zhou, 2020). Although one round of writing and feedback may be sufficient for understanding a learner's engagement with feedback, changes in responses toward feedback cannot be fully captured.

For data collection, various methods were used in previous studies. However, written reports or written verbalizations (e.g., journals, reflective accounts) or a combination of both verbal and written reports, and semi-structured interviews with both learners and their teachers (Lee et al., 2021) are all needed to make a study comprehensive. Classroom observations can also be used in the research to support the interview data (Storch, 2018). Even fewer studies have involved the methods summarized in Table 4. Because several studies mentioned the limitations of case studies where generalizations cannot be made (Shintani, 2016; Zheng et al., 2020a,b; Lee et al., 2021), future research should also involve more types of learners and take different learner variables into consideration. Teacher variables are also significant and more teachers' voices could be observed in future studies through interviews or written reports, such as diaries or reflective journals.

Most of the studies were conducted in China, which reveals a recent trend in Chinese education, particularly tertiary education where learners are viewed more as active agents in learning and their responses need to be considered to make teaching and learning more effective. This may lead to a break in the traditional idea that Chinese students learn without thinking critically. Most studies were located in ESL/EFL countries and they largely targeted international students. In the future research, more studies should be conducted comparing ESL/EFL learners across different countries.

### Theoretical Framework

The typology of learner engagement put forward by both Fredricks et al. (2004) and Ellis (2010) has been used by a few studies as their analytical or conceptual framework to provide systematic directions for research on learners' responses to TWF as the results show. However, its function is limited and if researchers expect to gain a more thorough understanding of learners' dynamic responses, other theories need to be integrated. As the results revealed, SCT was applied in six reviewed studies and appears to be an appropriate theoretical framework to investigate the complex relationship between TWF and students' responses (Storch, 2018; Lee, 2019a). This theory regards TWF as a method for mediating students' learning. Through the lens of ZPD, teacher feedback helps learners realize the gap between their current writing proficiency and the level they are expected to reach (Vygotsky, 1978). TWF is thus seen as an “opportunity” to higher intellectual levels when learners realize its value and actively engage with the feedback (Lee, 2019a). Although a few studies have investigated TWF based on SCT, the number is still very limited. Therefore, in future studies, researchers may consider exploring the mediating function of TWF and develop some L2 writing feedback activity models (e.g., Yu, 2013; Lee, 2014) by integrating the framework of learning engagement. However, few studies have explored students' responses to TWF by taking a SCT perspective. Thus, SCT can help theorize the framework of learner engagement. Other theories might also provide theoretical support and could be further explored.

### Learners' Responses and Influencing Factors

The review results have explicitly shown the complexity of learners' responses to TWF and influencing factors. This review summarizes the findings related to learners' responses based on the educational levels of the learners they were at as they participated in the research and the concepts of learner engagement but with a newly adapted one for the current review.

Results reveal that attitudinal responses are mainly comprised of preference, emotions, attitudes and these three aspects influence each other. Due to individual differences and contextual influences, learners' responses varied and seemed to be hard to predict but some trends were still noticed and provided directions for future research. Regarding the findings on students' preferences for TWF, it would be useful to investigate different feedback modes (e.g., comparison between computer-mediated feedback, face-to-face feedback, handwritten feedback, screencast feedback), feedback sources (e.g., TWF, peer feedback, automated feedback), feedback types (e.g., directness, focus, explicitness, correction, or other features), and timing (e.g., synchronous or asynchronous feedback). Emotions were found to be diverse, mainly positive, negative and neutral with neutral emotions receiving little attention. The results show most changes were from negative to positive or to neutral, while few studies reported changes from positive to negative or neutral, which can also happen; thus, the influencing factors need to be explored to help learners maintain positive responses to TWF. Emotions and cognition together mediate language learning (Swain, 2013). Learners have been found to show more rounds of interplay between attitudinal and cognitive responses and each interaction between these two aspects might promote the understanding and uptake of the feedback (Li and Curdt-Christiansen, 2020). Furthermore, cognitive responses were found to contain three aspects: noticing, processing and cognitive/metacognitive operations. Although the reviewed studies found the focus, speed, accuracy of noticing (e.g., lexical level), various processing operations and the outcome of process, why learners notice and process in those ways remains to be answered. Furthermore, learners also differed in their behavioral responses to TWF, including revision operations, feedback uptake and other related social behaviors, such as seeking help from peers or even deciding to marginalize oneself in a community (Mirzaee and Hasrati, 2014). For future research, attention could be focused on these extra social behaviors.

Similarly, influencing factors were also found to be diverse and varied in different contexts. Taking the sociocultural perspective, learner development is a movement from “interpersonal to intrapersonal communication” (Lantolf and Poehner, 2014, p. 45). In other words, human development is a social-to-individual progressive transition during which knowledge is “recreated, modified, and extended in and through collaborative knowledge building and individual understanding” (Wells, 1999, p. 89). Full understanding of the knowledge needs to go through a convention, taking two planes (first on the “intermental plane” and then the “intramental plane”) (Lantolf, 2000, p. 17). Learners develop themselves from object-regulation and other-regulation to self-regulation that they can “voluntarily organize and control (i.e., mediate) mental activity and bring it to the fore in carrying out practical activity in the material world” (Lantolf and Thorne, 2012, p. 62). This is why the factors were divided into inter-factors and intra-factors. Although the reviewed studies' findings and discussions offered various influencing factors, how these factors influence each other and what role they play in those contexts have not been thoroughly revealed. Taking the SCT standpoint, intra-factors play a more decisive role so more research emphasis is needed on these factors, e.g., students' feedback literacy (Yu and Liu, 2021). As Maas (2016) advises, more studies are needed to investigate the relationship between the learner autonomy and self-assessment and factors affecting students' needs of TWF because they can improve their writing and language proficiency most when they receive “text-specific, relevant, and clear” feedback that respects students' writing drafts and individual responsibility, and gives students enough space to decide how they respond to the feedback (Goldstein, 2006, p. 203). From the sociocultural viewpoint, TWF should be “tailored, graduated, and contingent” to satisfy individual needs and scaffold language learning when it is necessary (Li, 2020).

## Conclusion

Students' response to TWF is not a new topic; however, the term “learner engagement,” another term for learner responses to teacher feedback in L2 writing, appeared about 10 years ago. Following the trend to investigate students' responses within this framework, the present study has systematically reviewed studies on ESL/EFL learners' responses (i.e., engagement) to TWF from 2010 to 2021. The number of studies in this area is growing but still limited. Drawing upon the findings of this review, teachers may gain a better understanding of their feedback practices and possible reaction from students. Researchers may also be inspired to further explore student responses and the rationale behind them by taking a sociocultural perspective. One limitation of this study concerns the reliability of the data as only one coder was involved in the whole data collection and analysis procedure. Despite the possible omission of some articles and the publication bias (only articles in high-ranking journals were reviewed), this review can still provide insights into what has been investigated and underexplored in recent years.

## Author's Note

The terms learners' responses and learner engagement are used interchangeably in this review paper.

## Author Contributions

RY contributed to the design, data collection, data analysis of the study, and wrote the drafts of the manuscripts. LY guided RY through the writing process and helped RY revise the manuscripts. All authors have approved for publication of the review and are responsible for the review.

## Funding

This paper was part of the project of Research on Educating Excellent Foreign Language Teachers in the New Era supported by China National Social Science Found (#19BYY220).

## Conflict of Interest

The authors declare that the research was conducted in the absence of any commercial or financial relationships that could be construed as a potential conflict of interest.

## Publisher's Note

All claims expressed in this article are solely those of the authors and do not necessarily represent those of their affiliated organizations, or those of the publisher, the editors and the reviewers. Any product that may be evaluated in this article, or claim that may be made by its manufacturer, is not guaranteed or endorsed by the publisher.
