# Supplementary material for: ESL/EFL Learners' Responses to Teacher Written Feedback: Reviewing a Recent Decade of Empirical Studies
Source: Front Psychol. 2021 Oct 29;12:735101. doi: 10.3389/fpsyg.2021.735101 (PMC8586699; doi:10.3389/fpsyg.2021.735101)
Supplement: Supplementary file 1 [file Data_Sheet_1.docx]

**Appendix I Selected reviewed articles**

| Tertiary education | | | |
| --- | --- | --- | --- |
| Authors | Participants (research location) | Data collection methods | Results |
| Mahfoodh (2017) | 2 teachers + 8 EFL undergraduates (Yemen) | Student interviews | Complex relationship between learners’ emotional responses, TWF and success of revisions |
| Bakla (2020) | 33 intermediate EFL undergraduates and 6 interview undergraduates (lower, average, higher essay scores)  (Turkey) | Student interviews | Learner engagement with three online modes of TWF and influencing factors |
| Han & Hyland (2019a) | 2 teachers (1 native + 1 local) +  2 EFL undergraduates, with 1 from each teacher (high-achieving + under-achieving)  (China) | Student interviews, end-of-semester reflective accounts, teacher-student writing conferences, class observation and field notes | The emergence and change of learners’ emotional responses to teacher WCF in a general EAP course |
| Dikli & Bleyle (2014) | 1 teacher + 14 undergraduates (1.5 Generation)  (U.S.) | Automated feedback, questionnaire | Learners’ attitudinal responses of the application of an Automated Essay Scoring system and TWF |
| Xu, J (2021) | 311 EFL undergraduates  (China) | Student interviews, questionnaire, teachers’ descriptions of their online English writing teaching | Learners’ attitudinal responses to TWF, learning strategies and learners’ feedback-seeking orientation |
| Chong (2019) | 93 EFL undergraduates (low-achievers)  (China) | Student interviews, a reflection sheet | Learners’ attitudinal responses to e-feedback given by the teacher–researcher on Google Docs |
| Niu, Shan & You (2021) | 17 American students + 17 Chinese EFL students  (China) | Student interviews, written reflections, peer feedback | Students’ attitudinal responses to three feedback sources: Chinese teacher feedback, Chinese peer feedback, and American student feedback in a transnational writing activity |
| Mohammed & Alharbi (2021) | 56 low-intermediate EFL undergraduates  (Saudi Arabia) | Student interviews, students’ screencast recorded dialogue, students’ email messages and notes as they watched their screencast records | Students’ engagement in simultaneous peer dialogue and revisions |
| Turner (2021) | 25 EFL undergraduates  (Sweden) | A short reflective text, questionnaire | Complex relationship between student attitudinal responses to peer feedback and teacher feedback, academic achievement, and dialogic engagement with such feedback and learners’ literary disciplinary knowledge development |
| Mujtaba, Parkash & Nawaz (2020) | 90 EFL undergraduates  (Pakistan) | Questionnaire | Students’ attitudinal and cognitive responses to the teachers’ indirect coded correction feedback and affective short comments |
| Tang & Liu (2018) | 3 teachers + 64 EFL undergraduates  (U.S.) | Questionnaire | Relationship between learners’ writing performance, uptake, motivation and two feedback types (the Indirect coded correction feedback with and without short affective teacher comments) |
| Zhang, Chen, Hu & Ketwan (2021) | 117 EFL undergraduates  (Thailand) | Student interviews, questionnaire | Learners’ preferences on four types of written corrective feedback grammatical, lexical, orthographic, and pragmatic errors |
| Zhang & Cheng (2021) | 72 intermediate EFL undergraduates  (China) | Questionnaire | Learners’ attitudinal responses to comprehensive WCF on accuracy, syntactic complexity, and fluency |
| Han & Hyland (2015) | 1 teacher (native speaker of English) + 4 average EFL undergraduates  (China) | Student interviews, teacher interviews, retrospective verbal reports, class observation and observation notes | Learners’ cognitive, behavioral, and affective engagement with teacher WCF |
| Zheng, Yu, Wang & Zhang (2020) | 3 graduating EFL learners  (China) | Student interviews, teacher-student writing conferences | Learners’ affective, behavioural, and cognitive engagement with supervisor feedback on their thesis drafts |
| Saeed, Al Qunayeer, & AL-Jaberi (2021) | 1 teacher + 2 EFL postgraduates  (Malaysia) | Student interviews, written comments on feedback, | Learners’ engagement with supervisory feedback formulation on research proposal writing |
| Sherafati, Largani & Amini (2020) | 60 intermediate EFL undergraudates  (Iran) | Student interviews, automated feedback | Learners’ attitudinal responses on computer-mediated teacher feedback and computer-generated feedback |
| Zhang, X (2021) | 3 EFL undergraduates  (China) | Student interviews, weekly written reflections, teacher-student in-class conversations and field notes | Learners’ interaction with SFL-based (Systemic Functional Linguistics) teacher-written feedback |
| Secondary education | | | |
| Simard, Guénette & Bergeron (2015) | 49 ESL high school learners  (Canada) | Questionnaire | Learners’ engagement with the WCF received (direct or indirect provided in alternation) |
| Primary education | | | |
| Coyle, & Roca de Larios (2020) | 16 EFL learners  (unclear country) | Children’s audio recorded dialogues, written notes | Learners’ written corrective feedback (i.e., models) processing and uptake during a multi-stage writing task |

Note: TWF and writing drafts were two necessary and important sources in all the reviewed studies and the difference between each study was whether they were analyzed; thus, they are not mentioned in Appendix I. Other relevant methods are briefly summarized above.
